# Large-scale assessment of German dairy farmers´ personality and resulting ideas for improving veterinary consultancy

**DOI:** 10.1371/journal.pone.0277219

**Published:** 2022-11-23

**Authors:** Friedemann Adler, Amely Campe

**Affiliations:** Department of Biometry, Epidemiology and Information Processing, WHO Collaborating Centre for Research and Training for Health at the Human-Animal-Environment Interface, University of Veterinary Medicine Foundation, Hannover, Germany; University of Ilorin, NIGERIA

## Abstract

The here presented investigation was part of a cross-sectional study on dairy cattle health aiming to identify risk factors for animal health and welfare. Within this setting, we chose a social-science related approach to explore German dairy farmers’ personality composition in a variable-centered and person-centered approach. We have assessed German dairy farmers’ personalities based on the HEXACO model of personality in three representative regional samples. In total, 765 farm visits were conducted within two and half years (north n = 253; east n = 252; south n = 260). The proportions of returned questionnaires eligible for analysis (i.e., answered completely) were 71.54% (n = 181), 68.25% (n = 172) and 86.92% (n = 226) for the northern, eastern and southern samples, respectively. Variable-centered personality analyses revealed the farmers’ structure of personality to be similar within all samples. Overall, we found the farmers to be averagely emotional and agreeable, whereas the results indicate tendencies for a pronounced display of extraversion, conscientiousness and openness to experience. Compared to the German general population, dairy farmers appear to be more extraverted and open to experience. We could not deduce a subcategorization of farmers in different latent personality profiles in person-centered analysis. Our findings provide a basis for further evaluation of the farmers´ personality as an influencing factor for farm outcomes (e.g., productivity, animal health and welfare). Furthermore, we provide a glance and encourage interdisciplinary research to link personality information with communication theory aiming to enhance effectiveness of veterinary consultancy.

## Introduction

Today, more than ever before, veterinary specialists have to deal with increasingly strict requirements concerning the living conditions of farmed animals. Over the past years there is a highly visible trend observable in society showing that customers are no longer willing to accept a trade-off between effective farm outcomes and high technical and ethical standards concerning health and welfare of livestock. This results in the need to develop strategies for veterinary consultancy how to perform within that challenging area of conflict. The basis for those strategies is well-founded knowledge about factors influencing farm productivity as well as health and well-being of the animals.

Scientific research aiming to identify those influencing factors can look back on a remarkable amount of theory and findings. Over the past decades, related hypotheses have moved away from considering technical risk factors (e.g., housing, feeding) only but have more and more payed attention on the farmer himself an how decision makers as a person might impact farm-outcomes.

Since the early 1970s, when Seabrook [1] investigated the cowman’s effect on milk yield in dairy cattle, the psychological concept of personality has been widely known within animal science research. It has, among others, been used to explain farm outcomes by differences in farmers’ characters. Farm outcomes such as animal health, welfare and productivity have been shown to be associated with the farmers´ personality in multiple studies [2]. Furthermore, research, mainly within the human health sector, has recognized personality to be a potential predictor of patients´ communicational needs and preferences [3]. We argue that such approach might also be valuable in veterinary science in order to develop strategies within the field of “tailored veterinary consultancy”. According to that [4] have, for example, suggested an interdisciplinary approach to “know the customer”, a maxim that veterinarians have not yet commonly internalized [5].

To our knowledge, despite the aforementioned relevance of personality information, there is yet any large-scale assessment and description of the personalities of dairy farmers in Germany. However, knowing about the personality structure of German dairy farmers is essential to proceed within research helping to understand the farmers´ role in on-farm decision making, information perception during consultancy and resulting outcomes.

Contributing to this interdisciplinary field of research, we have assessed German dairy farmers´ HEXACO personalities in a large representative sample. The HEXACO model is a well-known and validated for personality assessment [6]. It assesses human personality based on six domains (Honesty-humility, Extraversion, Agreeableness, Conscientiousness and Openness to Experience). Each domain comprises four subordinate facets each adding additional personality information to the domains (see https://hexaco.org/scaledescriptions).

Data analysis in personality research is mainly based on two different analytical approaches. The more traditional **item-centered approach** uses personality traits (HEXACO domains) to predict other variables of interest (e.g., farm outcomes). It uses these traits individually or in combination [7]. Furthermore, the item-centered approach is based on the assumption that the relationship observed among personality traits and other variables are uniform within a given population [7].

In contrast, the **person-centered approach** assumes that there might be multiple unobserved subgroups within a study-population. Therefore, trait relations can differ among subgroups [7]. Person-centered approaches aim to identify these subgroups by means of different statistical approaches such as cluster analysis, latent-class-analysis (**LCA)** or latent profile analysis (**LPA**).

We aim to contribute to further evaluation of the effect of farmers´ personality on farm-outcomes. Our findings shall also provide a data basis for scientists aiming to link personality-information with communication theory to derive strategies on how to enhance effective communication in veterinary consultancy.

The research presented here is part of a cross-sectional epidemiological study that was carried out to assess dairy cattle health, productivity and farm management practices, to identify management-related risk factors affecting farm outcomes (i.e., health, wellbeing and productivity of dairy cows) and to develop modes of action for stakeholders within the dairy sector (e.g., farmers, vets). The overall aim was to improve farm outcomes based on these findings.

## Materials and methods

### Study area and design

The study “PraeRi” was conducted in three regions of Germany, which are characterized by intensive dairy farming. The northern region includes the federal states of Lower Saxony and Schleswig-Holstein. The eastern region consists of Brandenburg, Mecklenburg-Western Pomerania, Saxony-Anhalt and Thuringia. The southern region comprises Bavaria.

### Sample size determination and sampling technique

Multiple calculations were done to assure that data was assessed from adequate sample sizes. The challenge was to make a compromise between feasibility and necessity, taking into account different prevalence and quantitative parameters to be assessed. The final sample size was set to n = 250 farms per study region, assuming a disease prevalence of 50% on the farms, a precision of ± 5% and a 95% confidence interval. The calculation was performed using NCSS PASS Sample Size Software version 13.0.8 [8]. The actual number of farms located within the study regions differs between the samples. Consequently, the samples represent different proportions of the target population (north = 1.77%; east = 5.58%; south = 0.76%). We considered the three study regions of Germany separately, as the samples from these regions differ regarding general structure (i.e., average farm size, predominant mode of ownership [family business/cooperation]) and farm management (i.e., breed, access to pasture, animal housing [tie stall/free stall]). To assure a representative composition of the study population within each region regarding herd size, we categorized the farms into “small”, “medium” and “large” depending on the number of lactating cows kept at the date of sampling. The cut-offs were defined individually for each region.

### Data collection technique and measurement

Three teams of trained veterinarians conducted one-time farm visits for data collection. Each team was responsible for only one study region. The farm visits comprised a face-to-face interview and a set of on-farm observations (e.g., prevalence of disease, management aspects related to housing, feeding, breeding, etc.).

We used national and federal databases (HIT and LKV) to randomly select address information for potential participants. The teams then sent invitation letters to the farms. Those farmers willing to participate contacted the veterinary team responsible for their study region, which, in turn, scheduled the farm visits.

A modified version of the evaluated 24-item Brief HEXACO Inventory [9] was used for personality assessment. We excluded the honesty-humility domain from the questionnaire due to concerns of our research partners about some statements (e.g., “I would like to know how to make lots of money in a dishonest manner”) being potentially compromising to the farmers. Consequently, the final personality questionnaire consisted of 20 self-descriptive statements. Farmers could rate their consent with each statement on a five-point Likert scale ranging from 1 = “fully disagree” to 5 = “fully agree” with a central neutral response option and verbal anchors at all scale points. Scale points were presented on a horizontal line. Consent had to be marked with a cross by the farmers. We exclusively assessed the personality data from those interview partners who were decision makers on the farms to be able to link farmers´ personality information and the factual on-farm situation in intended further analyses. After the completion of the face-to-face interview, the farmer filled in the personality-questionnaire by his/herself and enclosed it in a sealable envelope (attached to the questionnaire) to meet confidentiality requirements. The only indication of farm affiliation was a pseudonymized farm-ID noted on the envelope to ensure the proper assignment of the personality information to the other farm-data in the study database. The study teams sent the envelopes containing the personality questionnaires to the first author who then entered the data into a project-specific online database.

### Data quality control

We created standard operating procedures prior to the assessments to minimize observer bias [10]. We piloted the assessments before the start of the regular data collection. Pilot testing was carried out on three farms in each study region. It provided the researchers with the possibility to get used to the procedures and get an impression of how famers reacted to the personality assessment. The experiences were discussed later on, to refine and harmonize the assessment approach to reduce observer bias. All pilot farms were excluded from the analysis.

### Ethical considerations

Ethical standard requirements of the personality questionnaire were discussed within the study group. All researchers were aware of the need to strictly meet confidentiality requirements during data assessment. A specific standard operation procedure (including a standardized introduction text) was developed to ensure that all farmers were comprehensively informed about the purpose of the personality questionnaire against the background of the studies´ context. In addition to the aforementioned (i.e., leaving farmers alone for rating of the questions, sealable envelopes, data management performed by one researcher only) and strict interdiction of any probing in case farmers would indicate not to participate in the personality assessment ensured conformity of the assessment with ethical standards and codes of best practice. Ethical approval (Approval code TiHo-REC_07_22) was granted by the Research Ethics Committee of the University of Veterinary Medicine Hannover, Foundation. The committee checked and confirmed methodology concerning scientific approach, funding and content and design of questionnaires. Furthermore, consent of the participants concerning voluntary participation and privacy policy were reviewed by the committee.

### Data analysis

In this paper, we operate both, item-centered and person-centered analytical approaches and report on the structural composition of German dairy farmers’ HEXACO personality.

The data was analyzed separately for the regional samples. We performed analyses using SAS^®^ software, version 9.4 of the SAS^®^ system for Windows^®^ (copyright © 2002–2012 SAS Institute Inc., Cary, NC, USA). Only fully completed questionnaires were included.

A **variable-centered approach** was applied to describe the status quo of German dairy farmers’ personality. Data related to reverse-worded questionnaire statements (Table 1) were recoded contrariwise. Consequently, a higher rating on the Likert scale always indicated a stronger display of a facet within a persons’ personality. To obtain personality information for each of the five domains (e.g., emotionality [HE]), we calculated the median from the Likert scale ratings (1–5) of the related facets (e.g., HE1, HE2, HE3, HE4) per individual farmer. For these median values, distribution was analyzed descriptively using measures of central tendencies. Averaged median values greater than three indicated a pronounced display of a personality domain within a sample (the higher the value, the stronger the domain’s weight and contrariwise).

**Table 1:** Structure of the 24-item BHI used for personality assessment.

Domain	Item	Facet	Statements in questionnaire^1^
Honesty-Humility (HH)^2^	HH1	Sincerity	I find it difficult to lie.
	HH2	Fairness	I would like to know how to make lots of money in a dishonest manner.
	HH3	Greed-Avoidance	I want to be famous.
	HH4	Modesty	I am entitled to special treatment.
Emotionality (HE)	HE1	Fearfulness	I am afraid of feeling pain.
	HE2 (R)	Anxiety	I worry less than others.
	HE3 (R)	Dependence	I can easily overcome difficulties on my own.
	HE4	Sentimentality	I have to cry during sad or romantic movies.
Extraversion (HX)	HX1(R)	Social Self-Esteem	Nobody likes talking with me
	HX2	Social Boldness	I easily approach strangers.
	HX3	Sociability	I like to talk with others.
	HX4 (R)	Liveliness	I am seldom cheerful.
Agreeableness (HA)	HA1 (R)	Forgiveness	I remain unfriendly to someone who was mean to me.
	HA2 (R)	Gentleness	I often express criticism.
	HA3	Flexibility	I tend to quickly agree with others.
	HA4	Patience	Even when I am treated badly, I remain calm.
Conscientiousness (HC)	HC1	Organization	I make sure that things are in the right spot.
	HC2 (R)	Diligence	I postpone complicated tasks as long as possible.
	HC3	Perfectionism	I work very precisely.
	HC4 (R)	Prudence	I often do things without really thinking.
Openness to experience (HO)	HO1	Aesthetic appreciation	I can look at a painting for a long time.
	HO2 (R)	Inquisitiveness	I think science is boring.
	HO3	Creativity	I have a lot of imagination.
	HO4	Unconventionality	I like people with strange ideas.

^1^All statements were rated on a 5-point Likert scale.

^2^Domain was excluded from the assessment.

(R) = Reverse items. Ratings were recoded reversely for analysis.

To investigate the possibility of deducing personality profiles from the data, a **person-centered approach** was applied. We conducted a latent class analysis (**LCA**) by means of PROC LCA [11]. The analysis utilized the recoded Likert scale ratings per facet as the input data. As recommended by [12], baseline models were fitted for predefined numbers of 1 to 7 latent classes. We used default values for the maximum number of iterations (n = 5000) and absolute deviation (0.000001). One hundred percent of the seeds were required to be associated with the maximum likelihood (**ML**) solution using 10 randomly selected starting values to consider a model for further evaluation. The Akaike information criterion (**AIC**) and Bayesian information criterion (**BIC**) were indicators of relative model fit. We considered entropy as a measure for classification certainty. Furthermore, attention was paid to parsimony, content-related interpretability and spuriousness to identify a model that optimally represented the data [12].

## Results

In total, 765 farm visits were conducted within a period of approximately two and half years (north n = 253; east n = 252; south n = 260). The personality assessment resulted in 71.54% (n = 181), 68.25% (n = 172) and 86.92% (n = 226) of fully answered questionnaires in the northern, eastern and southern regions, respectively.

### Structural composition of the farmers’ HEXACO personality

#### Emotionality

**Fig 1:**
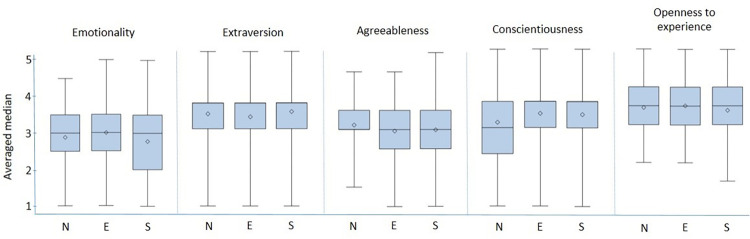
Distribution of median ratings per the HEXACO personality domains of dairy farmers for three different regions of Germany (n_north_ = 181; n_east_ = 172; n_south_ = 226). N = north; E = east; S = south.

**Fig 2:**
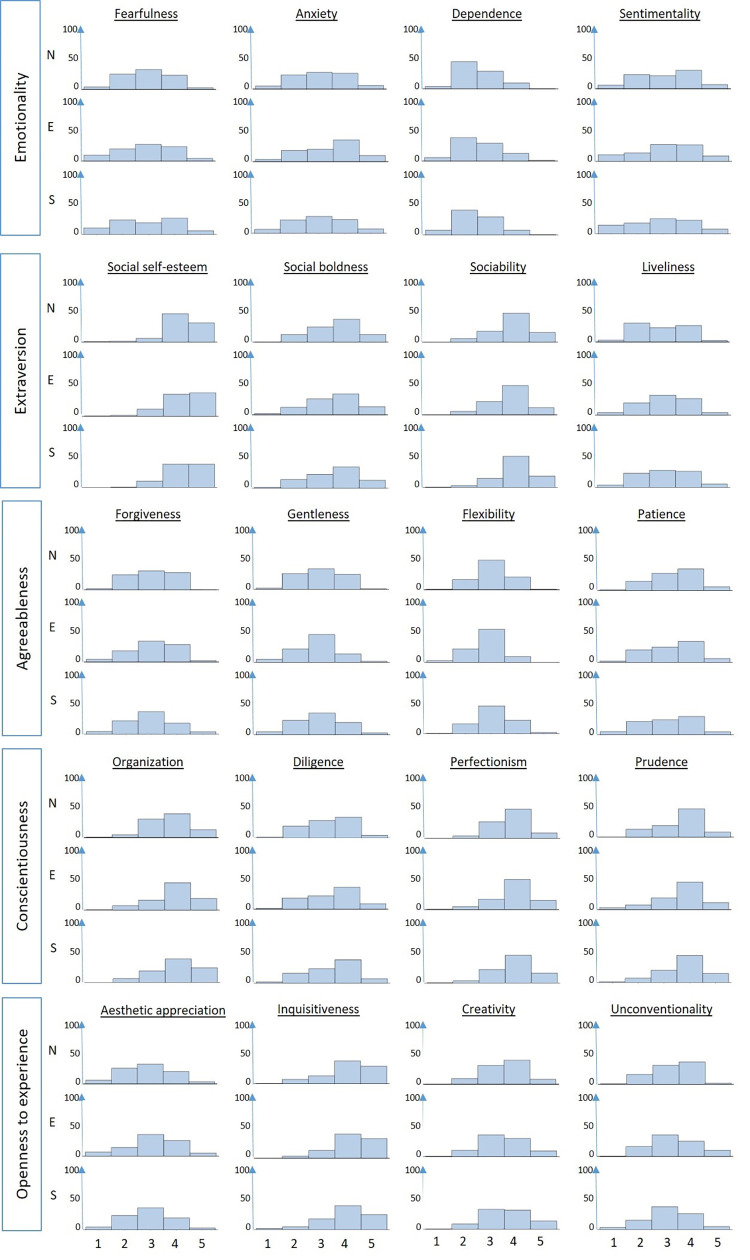
Distribution of the Likert scale ratings of HEXACO personality facets for dairy farmers in three different regions of Germany (n_north_ = 181; n_east_ = 172; n_south_ = 226).

The distributions of the averaged domain medians (Fig 1) showed the farmers to be averagely emotional across all three regions. Regarding the facets related to the emotionality domain (Fig 2), the distribution of the ratings was relatively homogenous among the samples. Remarkably, eastern farmers seemed to be more anxious than farmers from the north and south, as indicated by the largest single proportion (51.91%; n = 95) disagreeing with the related statement compared to proportions of 36.36% (n = 72) and 34.43% (n = 84) in the north and south, respectively. However, the largest single proportions of northern (30.50%; n = 61) and southern famers (32.02%; n = 81) rated the related statement neutrally.

#### Extraversion

Farmers from all three regions were characterized by pronounced extraversion, as indicated by averaged domain medians of 3.79 (SD = 0.61), 3.73 (SD = 0.64) and 3.83 (SD = 0.64) in the north, east and south, respectively. Regarding the related facets, all the samples were characterized by pronounced social self-esteem, social boldness and sociability. While the biggest proportions of farmers from the eastern and southern samples obtained neutral ratings for the “liveliness” statement (37.84%; n = 70 and 31.71%; n = 78, respectively), the biggest single proportion of northern farmers (39.20%; n = 78) agreed or strongly agreed to be seldom cheerful, thus indicating lower levels of liveliness in comparison.

#### Agreeableness

Only northern farmers expressed a slightly pronounced agreeableness (averaged median = 3.11; SD = 0.60), while southern and eastern farmers indicated an average display of that domain. While the biggest proportions of farmers from all three regions obtained neutral ratings for “forgivingness”, “gentleness” and “flexibility”, the distribution of rating within the “patience” facet was skewed to the right, thus indicating farmers’ pronounced patience in all regions

#### Conscientiousness

Farmers from all samples showed pronounced conscientiousness, obtaining averaged medians of 3.60 (SD = 0.60), 3.77 (SD = 0.63) and 3.75 (SD = 0.67) in the northern, eastern and southern regions, respectively. At the facet level, farmers indicated being diligent, organized, perfectionistic and prudent. The proportion of northern farmers who considered themselves to be organized (60.10%; n = 119) was lower than the 71.35% (n = 132) and 72.06% (n = 178) within the eastern and southern regions.

#### Openness to experience

Farmers in all regions indicated being open to experience. Between 73.06% (n = 179; south) and 80.54% (n = 149; east) of the participants considered themselves inquisitive. The biggest single proportions of farmers from all regions (north: 52.28%, n = 103; east: 44.81%, n = 82; south: 53.06%, n = 130) claimed to have a lot of imagination, thus considering themselves to be predominantly creative.

### Personality profile analysis

Table 2 reports the absolute and relative model-fit indicators derived from SAS^®^ software PROC LCA baseline model selection. In any baseline model estimation with the number of classes being greater than one, 100% of the seeds were associated with the maximum likelihood solution. Hence, evaluating the relative model fit was not applicable. In conclusion, the analysis did not reveal an indication of the possibility of subgrouping farmers regarding personality profiles.

**Table 2:** Person-centered analysis of personality profiles: Absolute and relative model fit of SAS® software PROC LCA baseline model estimation by region.

Number of classes	G-squared	AIC	BIC	LL^1^	Entropy	Seeds associated with ML solution (%)
**North**						
1	6999.90	7159.89	7415.77	-4440.87	1	100
2	6778.51	7100.51	7615.47	-4330.18	0.81	10
3	6580.44	7064.44	7838.48	-4231.15	0.90	10
4	6441.94	7087.94	8121.06	-4161.90	0.92	10
5	6327.22	7135.22	8427.41	-4104.54	0.94	10
6	6223.50	7193.50	8744.77	-4052.68	0.95	10
7	6143.51	7275.51	9085.86	-4012.68	0.97	10
**East**						
1	6994.11	7154.11	7405.91	-4382.42	1	100
2	6672.79	6994.79	7501.53	-4221.76	0.95	20
3	6511.38	6995.38	7757.07	-4141.06	0.91	10
4	6377.91	7023.91	8040.55	-4074.32	0.94	10
5	6279.83	7087.83	8359.42	-4025.28	0.94	10
6	6209.61	7179.61	8706.15	-3990.18	0.96	10
7	6088.89	7220.89	9002.38	-3929.82	0.97	10
**South**						
1	9281.98	9441.98	9715.63	-5866.03	1	100
2	8880.81	9202.81	9753.52	-5665.45	0.90	40
3	8652.65	9136.65	9964.42	-5551.37	0.92	10
4	8514.77	9160.77	10265.60	-5482.43	0.91	10
5	8351.16	9159.15	10541.05	-5400.62	0.92	10
6	8240.92	9210.92	10869.88	-5345.50	0.95	10
7	8155.45	9287.45	11223.47	-5302.77	0.96	10

^1^ Log Likelihood

## Discussion

Discussing the structural composition of farmers´ personality and comparing the findings of different studies has to consider that personality assessments can be based on a variety of different analytical approaches concerning underlying theoretical frameworks and questionnaires. Furthermore, selection bias [10] as a result of the study setting can be an issue. However, there is large overlap between the different approaches to personality assessment, and selection bias is an issue for all self-report assessments based on voluntary participation. Hence, we consider our data eligible for contextual interpretation. The 24-item BHI [9] is a specialized tool for settings in which the use of a full-length inventory is not applicable such as “PraeRi” due to the complexity of on-farm assessments and number of questionnaire-items (i.e., 376 items per farm). We expected it to be a challenge for participants to be focused on answering the personality questionnaire on the day of the farm visit due to the many unusual impressions they had to face on that day. The 24-item BHI was constructed for exactly such situation [9]. [9] was able to show that, although characterized by relatively low alpha reliability (.34 to .72), the inventory re-estimates the construct validity of the original HEXACO-PI-R with relatively great accuracy. Two-month test-retest stability, and self-other agreement indicate only modest loss of validity compared to the full-length inventory [9].

Item-centered analysis has revealed German dairy farmers to be characterized by pronounced extraversion, conscientiousness and openness to experience. Furthermore, results indicated slightly pronounced agreeableness among farmers. Due to the HECAXO scale description (https://hexaco.org/scaledescriptions), extraverted persons feel positive about themselves. They are characterized by confidence in leading situations or when they have to address other people. They enjoy themselves in social contexts and feel enthusiasm and energy. High scores on conscientiousness indicate to be well organized as well as to be accurate and perfectionistic when performing tasks. People scoring high on conscientiousness also tend to be deliberate and careful decision-makers. Pronounced openness to experience is related to being inquisitive about various domains of knowledge as well as taking interest in unusual ideas or people.

Transferred to the current situation and working conditions of dairy farmers in Germany nowadays, it becomes apparent that all these traits can be considered essential to run a profitable business within the dairy cow sector. Farmers have to be creative as concerns the development and entrepreneurial orientation of their businesses. They have to be proactive communicators as they are confronted with multiple stakeholders within the sector (e.g., veterinarians, consultants, employees) which do all have their individual tasks and requirements. Finally, proceeding technical innovations require to be well informed and inquisitive about innovations which enter the dairy cow business. Future research should refine these results. An interesting question to address would be whether personality measures are able to predict farm success or the ability to sustain one´s position in the aforementioned environment of diverse interest of stakeholders and political requirements, farmers have to address.

Although this is the first time that German dairy farmers’ personality has been assessed based on the HEXACO framework, there is other item-centered research on the personality of dairy farmers or stockpeople. To relate our findings with those of former investigations within the animal science sector, we exemplarily chose two studies that refer to the five-factor model of personality (**FFM**). The FFM has been the basis for developing the HEXACO model. Both of these studies also used conventional, validated inventories for data assessment. [13] assessed the personality of dairy stockpeople in Northern Ireland. Our findings differ with regard to the display of “extraversion” and “conscientiousness”. While German dairy farmers showed a pronounced display of these two domains, [13] found contrary results.

Furthermore, [14] analyzed FFM personalities in a sample of Czech dairy stockpeople. The highest domain mean values were observed for “conscientiousness”, which is consistent with our findings. However, concerning farmers’ openness to experience, our results differ from those of [14], as German dairy farmers showed pronounced openness, which was not the case in their study.

To evaluate whether the personality of German dairy farmers might differ from that of the German general population, we obtained reference data published by [13]. They provide domain mean values for a sample of n = 2100 people who had answered an online form of the HEXACO-100 inventory for the purpose of self-exploration. Compared to this reference data, German dairy farmers do not differ from the German general population in terms of average emotionality as well as pronounced conscientiousness and openness to experience. However, our results indicate farmers to be more agreeable and extraverted than the general population.

The comparison of results between our data and the findings reported from other, exemplarily chosen, samples [13–15] reveals heterogeneity of findings. Tackling the question of the reasons for this observation has to consider multiple factors. Technical aspects related to data assessment have to be considered. The questionnaires used for data assessment differ between all considered studies. Furthermore, studies are not directly comparable concerning farmers´ social and entrepreneurial environments. We hypothesize, that factors such as farm type (family business/ cooperation), farm size (importance of dairy cattle for the entire income, degree of industrial automation) or even factors such as farm localization (political environment, prosperity of the geographical region) might influence the way people rate statements presented during personality assessment.

It becomes clear, that the answer to the essential question, whether farmers differ in their personality composition, requires harmonized data assessments. Only in case potential bias is controlled as strictly as possible allows for a robust interpretation of results in an overall context. This, in turn, requires enhanced correspondence and cooperation between research groups with the aim to validate personality assessments in comparable study populations.

The most common approaches to person-centered personality analysis include cluster analyses and latent profile analyses (**LPA**; [7]). However, we used LCA. LCA is a specialized tool for ordinal data [12]. The main advantage of LCA is that it is a model-based technique, which provides various statistical indicators that help to find the optimal model from a set of alternatives [7]. Furthermore, LCA provides the possibility of output values (i.e., posterior probabilities) for individuals (i.e., farmers), indicating the likelihood of a person being a member of a latent class [7, 12]. This information can then be used in subsequent analyses relating personality information to other factors of interest.

According to the literature, we expected to find three to five latent classes (i.e., personality profiles). Studies identifying three profiles within the FFM have consistently labeled these profiles as “resilient”, “overcontrolled” and “undercontrolled” [16–18]. Studies identifying more than 3 profiles mostly use some variation of these names [19–21]. Evaluating these results should consider that [20] argue that the consistency of prototypes was “far from being perfect” across different studies.

Contrary to the FFM, there has only been limited person-centered research within the HEXACO framework, with mixed results [7]. [22] were the first to use person-centered cluster analysis on HEXACO data, and they concluded that there was no clear clustering. Hence, this result is in correspondence with our findings. However, [7] identified five profiles by means of latent profile analysis. [23] used a 20-item short FFM inventory and added four additional items for “honesty-humility” to approximate the HEXACO framework. Using LPA, the authors identified four profiles.

It becomes clear that there is yet any common consensus as regards research on latent profiles within the different personality frameworks.

To our knowledge, the current study is the first time that the 24-item BHI [9] has been used in a person-centered approach. Furthermore, we chose LCA as a tool to extract potential profiles. Hence, the method impedes the direct comparison of our findings to those of other studies. However, inventories of comparable size and form have already been used successfully to extract profiles from personality data, including samples other than from the general population (e.g., prisoner sample; [20]). In conclusion, person-centered research on personality is characterized by heterogeneity regarding methodology and outcomes. Our results fit in this picture contributing to that heterogeneity of findings. Hence, further research is required to review and link existing results based on different personality inventories and analytical approaches.

We argue that further related research might have the potential to add valuable information to the question of how to strengthen beneficial interdisciplinary cooperation between social sciences and veterinary medicine. Veterinary specialists, have to deal with human personalities in daily practice. Livestock owners always function as intermediates between veterinarian and actual patients (i.e. animals).

As already mentioned before, it is generally plausible that personality and communication styles are closely linked [9]. Researchers such as [3] argue the application of personality type in human healthcare communication. Related research has established approaches that link patients´ personality with communication theory. The idea behind is that different personal dispositions in patients require adapted communicational strategies within consultation to ensure (1) better client satisfaction, (2) effective exchange of information and, in consequence, (3) enhanced compliance with medical advice. One example for a concept providing health professionals with practical tool on how to adapt communication to the individual needs of patients is the Flex Care^®^ model [24]. The general concept of Flex Care^®^ is that consultants should identify both their own preferred communication style and the one of the person they have to address. Consultants should then, if necessary, adapt (flex) their communication style to match that of the client. What is, so far, missing is an evidence-based link between the established and validates measures of personality (HEXACO, FFM) and such practical tools for specialists in the field. Assessing farmers´ communication style and establishing a link between communication and personality was beyond the scope of the here presented study. Therefore, we strongly recommend further interdisciplinary research contributing to the development of such practical tools to enhance the quality and success of veterinary consultancy.

## Conclusions

We provide primary information and a data basis on the status quo of German dairy farmers’ HEXACO personality. A variable-centered approach revealed pronounced levels of extraversion, conscientiousness and openness to experience. Those traits can be considered key qualifications against the background of entrepreneurial challenges German dairy farmers have to face during their daily business. Person-centered analyses did not lead to any latent personality-profiles which conforms to the heterogeneity of findings within this research area. Future interdisciplinary research is encouraged to further explore comparability of different personality concepts and assessment tools. Open communication and interdisciplinary work among scientists is considered essential to further explore the role of farmers´ personality as influencing factor for farm outcomes and its value concerning enhanced veterinary consultancy.
